# The Good of Medical Societies

**Published:** 1883-04-20

**Authors:** 


### *THE GOOD OF MEDICAL SOCIETIES.*

In addition to the instruction and mental improvement resulting from Medical Associations and Societies may be mentioned the social advantages to the members of the profession. It is often charged that Doctors are not friendly and sociable as among themselves. It must be admitted that in many communities the charge is correct. Yet it ought not to be so, and it has been observed that in places where the members of the profession meet in medical societies, and talk familiarly of their professional experiences, and interchange views in regard to their cases, that kind and social feelings always prevail, and that Doctors mingle freely together, finding both pleasure and profit in each others' society.

